# Supplementary material for: Causal Relationship between Aging and Anorexia Nervosa: A White-Matter-Microstructure-Mediated Mendelian Randomization Analysis
Source: Biomedicines. 2024 Aug 16;12(8):1874. doi: 10.3390/biomedicines12081874 (PMC11351342; doi:10.3390/biomedicines12081874)
Supplement: Supplementary file 1 [file biomedicines-12-01874-s001.zip › Supplementary Figure/Figure S8/Leaveoneout plot of MR analysis for TL to ubm-b-1910.pdf]

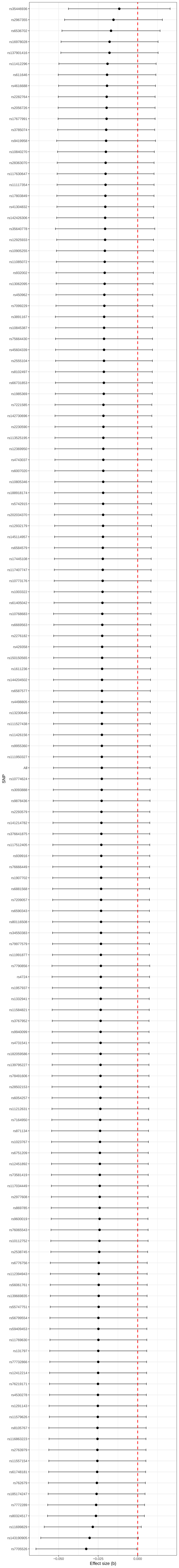
rs35446936
rs2967355
rs6536702
rs16978028
rs137901416
rs11412296
rs611646
rs4616688
rs2282764
rs2056726
rs17677991
rs3785074
rs9419958
rs10840270
rs28363070
rs117630647
rs11117354
rs17803849
rs41304832
rs142426306
rs35640778
rs12925933
rs10905255
rs11085072
rs932002
rs13062095
rs450962
rs7099229
rs3891167
rs10845387
rs75664430
rs45604339
rs2555104
rs8102497
rs66731853
rs1985369
rs7221585
rs142730696
rs2230590
rs113525195
rs12369950
rs4743037
rs6007020
rs10805346
rs188918174
rs5742915
rs202034370
rs12932179
rs145114957
rs6584579
rs17445108
rs117407747
rs10773176
rs1003322
rs61405042
rs10768883
rs6669563
rs2276182
rs429358
rs150150565
rs1611236
rs144204502
rs6587577
rs4498805
rs13230646
rs111527438
rs11426156
rs9955360
rs111950327
A8
rs10774624
rs3093888
rs9878436
rs2293579
rs141214782
rs376641875
rs117512405
rs939916
rs76666449
rs1907702
rs6881568
rs7209057
rs6590343
rs80116508
rs34550383
rs79977579
rs11991877
rs7790856
rs4724
rs1957937
rs1332941
rs11584821
rs3767952
rs9940099
rs4731541
rs182059586
rs139795227
rs78491606
rs28502153
rs6054257
rs11212631
rs7164950
rs871134
rs1023767
rs6751209
rs12451892
rs73581419
rs117034449
rs2977608
rs869785
rs9600019
rs76065543
rs10112752
rs2538745
rs6776756
rs112394943
rs56061761
rs139669835
rs55747751
rs56799554
rs59409453
rs11769630
rs131797
rs77732866
rs12412214
rs76219171
rs4530278
rs1291143
rs11579626
rs8105767
rs116863223
rs2763979
rs11557154
rs61748181
rs762679
rs185174247
rs7772289
rs80324517
rs11699829
rs143190905
rs7705526
SNP
-0.050
-0.025
0.000
Effect size (b)
